# Relationship Between Seizure Frequency and Functional Abnormalities in Limbic Network of Medial Temporal Lobe Epilepsy

**DOI:** 10.3389/fneur.2019.00488

**Published:** 2019-05-08

**Authors:** Hang Joon Jo, Daniel L. Kenney-Jung, Irena Balzekas, Kirk M. Welker, David T. Jones, Paul E. Croarkin, Eduardo E. Benarroch, Gregory A. Worrell

**Affiliations:** ^1^Department of Neurology, Mayo Clinic, Rochester, MN, United States; ^2^Department of Neurologic Surgery, Mayo Clinic, Rochester, MN, United States; ^3^Department of Radiology, Mayo Clinic, Rochester, MN, United States; ^4^Department of Psychiatry and Psychology, Mayo Clinic, Rochester, MN, United States

**Keywords:** medial temporal lobe epilepsy, partial seizure, functional magnetic resonance imaging, network-based statistics, limbic system

## Abstract

**Background:** We compared resting-state functional connectivity (RSFC) among limbic and temporal lobe regions between patients with medial temporal lobe epilepsy (mTLE) and healthy control subjects to identify imaging evidence of functional networks related to seizure frequency, age of seizure onset, and duration of epilepsy.

**Methods:** Twelve patients with drug-resistant, unilateral medial temporal lobe epilepsy and 12 healthy control subjects matched for age, sex, and handedness participated in the imaging experiments. We used network-based statistics to compare functional connectivity graphs in patients with mTLE and healthy controls to investigate the relationship between functional connectivity abnormalities and seizure frequency.

**Results:** Among mTLE patients, we found functional network abnormalities throughout the limbic system, but primarily in the hemisphere ipsilateral to the seizure focus. The RSFCs between ipsilateral hypothalamus and ventral anterior cingulate cortex and between ipsilateral subiculum and contralateral posterior cingulate cortex were highly correlated with seizure frequency.

**Discussion:** These findings suggest that in mTLE, changes in limbic networks ipsilateral to the epileptic focus are common. The pathological changes in connectivity between cingulate cortex, hypothalamus and subiculum ipsilateral to the seizure focus were correlated with increased seizure frequency.

## Introduction

The limbic system of the brain is a complex network of structurally and functionally linked anatomic regions (1). Among its components are areas in anteromedial temporal lobe including the amygdala, hippocampal complex, and entorhinal cortex, as well as the cingulate cortex. These areas are interconnected with each other via the thalamus (particularly the anterior thalamic and medial dorsal nuclear groups as well as the midline thalamic nuclear group), and project to the hypothalamus and midbrain, and anteromedial temporal lobe. The limbic system is an important component of the systems subserving emotion, behavior, and memory. In patients with medial temporal lobe epilepsy (mTLE), perturbations of the limbic system often result in in debilitating comorbidities and functional impairments in addition to the direct consequences of seizures.

The role of the limbic system in epilepsy is well-described, with mTLE frequently involving limbic structures and the pathological hallmark of hippocampal sclerosis (2–4). The limbic system's physiologic ability to produce and propagate synchronized activity during normal cognition makes the limbic system an ideal environment for the propagation of pathological synchronization during a seizure (5). Given the role of the limbic system in long-term recall and in emotion, it has been postulated that the high prevalence of memory, mood, and affective symptoms among patients with mTLE correlates with a disruption of normal limbic function (5–7).

Networks throughout the brain are altered in epilepsy. Network changes have been associated with cognitive decline, seizure onset zone locations, and surgical outcomes (8, 9). For example, increased thalamic “hubness” (the importance of a node in a network) prior to anterior temporal lobectomy surgery is associated with increased risk of seizure recurrence after surgery (10). Effective connectivity inferred by dynamical causal modeling has suggested that strengthened connectivity between the hippocampus and parahippocampal gyrus is associated with poor seizure control in TLE (11). Despite promising associations between limbic circuitry and clinical features, the contribution of limbic connectivity to actual seizure burden remains unclear.

To identify resting-state functional connectivity (RS FC) features associated with seizure frequency, we assessed limbic and temporal RS FC in mTLE patients and healthy controls. We used network-based statistics to identify functional connectivity abnormalities in the limbic system, and to find a relationship between those abnormalities and seizure frequency. By identifying the networks underlying clinical presentations of epilepsy, we may better target neuromodulatory approaches to the circuit components either potentiating or regulating epileptic networks.

## Materials and Methods

### Participants and Clinical Scores

Consecutive mTLE patients who underwent comprehensive epilepsy evaluations including EEG monitoring at the Mayo Clinic Epilepsy Center were identified from an epilepsy research database. Twelve patients (five females) with unilateral mTLE (Table 1) and 12 healthy control subjects (Table 2), matched to the mTLE subjects by age, gender, and handedness, were studied. The control subjects were free of neurological and psychological disease. All participants provided written, informed consent in accordance with research protocols approved by the institutional review board of Mayo Clinic. We collected the numbers of seizures per month during the 3 months preceding the MRI scan as a quantitative score of disease burden. Structural MRI data were reviewed for all subjects to assess for neuroradiological abnormalities. A board certified psychiatrist (PEC) retrospectively reviewed all clinical records to ascertain if subjects had a co-occurring psychiatric disorder. In mTLE group, there were 8 subjects with neuroradiological abnormalities and 9 subjects with psychiatric disorders.

**Table 1:** Demographic information for participants with medial temporal lobe epilepsy (mTLE).

**Subject index (*N* = 12)**	**Video-EEG diagnosis; seizure onset zone**	**Handedness**	**Age range (year)**	**Epilepsy duration (year)**	**Seizure frequency (number of seizures per months)**	**Neuroradiological abnormality in the structural MRI assessment**	**Psychiatric symptom**	**Psychotropic medication**
01	Right mTLE	Right	60–69	50	0.1	None	Depression, Anxiety	Citalopram
02	Left mTLE	Right	30–39	16	8	None	Remote history of alcohol and cannabis abuse (8–10 years before presentation)	None
03	Left mTLE	Right	40–49	42	8	Stable non-specific foci of increased T2/FLAIR signal in the subcortical left frontal lobe white matter	None	None
04	Right mTLE	Right	20–29	28	30	Right mesial temporal sclerosis	Anxiety	Citalopram, sertraline
05	Left mTLE	Right	10–19	16	4	None	Insomnia	Clonazepam
06	Left mTLE	Right	30–39	32	24	Left hippocampal atrophy	Depression, ADHD	Mixed amphetamine (Adderall XR)
07	Left mTLE	Right	20–29	12	3	Mild leukoaraiosis	Anxiety	None
08	Left mTLE	Left	20–29	7	2	T2 hyperintensities suggestive of migraine	Depression, nicotine-use disorder	Citalopram
09	Left mTLE	Right	20–29	1	20	None	Nicotine-use disorder (smokeless tobacco)	None
10	Right mTLE	Right	50–59	2	3	Right temporal encephalocele	None	None
11	Left mTLE	Left	20–29	22	1	Left hippocampal atrophy	None	None
12	Left mTLE	Right	30–39	6	16	Nonspecific T2 hyperintensities	Anxiety	Alprazolam

*Twelve healthy control subjects were matched for age, sex, and handedness (N = 12, mean age difference = 0.00; p > 0.99). The indirectly identifiable patient data (gender, exact age, exact seizure onset age) were removed according to editorial guidance*.

**Table 2:** Demographic information for healthy control subjects (five females).

**Subject index (*N* = 12)**	**Handedness**	**Age range (year)**	**Neuroradiological abnormality in the structural MRI assessment**	**Psychiatric symptom**	**Psychotropic medication**
01	Right	50–59	None	None	None
02	Right	30–39	None	None	None
03	Right	40–49	None	None	None
04	Right	20–29	None	None	None
05	Right	10–19	Possible pineal cyst	None	None
06	Right	30–39	None	None	None
07	Right	20–29	None	None	None
08	Right	20–29	None	None	None
09	Right	20–29	None	None	None
10	Right	50–59	None	None	None
11	Left	20–29	White matter hyperintensities	None	None
12	Left	20–29	None	None	None

### Image Acquisition

Anatomical and functional MRI images were acquired in all subjects on a Siemens 3T Magnetom Skyra system using a 32-channel array head coil and tetrahedron-shaped foam pads to minimize head movement. High-resolution structural whole-brain images were acquired using a T1-weighted sequence with 0.5 × 0.5 × 1.2 mm^3^ resolution, TR = 2.3 s, TI = 0.9 s, TE = 1.96 ms, and FA = 9°. Subjects were instructed to keep their eyes open during which resting-state (RS) functional magnetic resonance imaging (FMRI) data were acquired by using a gradient echo-planar sequence sensitive to blood oxygenation level-dependent contrast with 3.28 × 3.28 × 3.3 mm^3^ resolution, 50 slices, TR = 2.9 s, TE = 30 ms, FA = 90°, and total acquisition time of 464 s.

### Preprocessing of Imaging Data

Preprocessing of all imaging data was conducted using the Analysis of Functional NeuroImages (AFNI) software package (http://afni.nimh.nih.gov). The RS FMRI data were preprocessed and denoised by the standard protocol of the AFNI package (12–14). Using the robust non-linear warping function of the AFNI package, all T1 anatomy data were registered to the MNI152-T1-2009c atlas of the Montreal Neurological Institute and linearly resampled in the 1 mm isocubic grid space. The EPI data were aligned to the T1 images, and then warped to the template brain space along with their T1 images. The registration results for the image data of all subjects were visually inspected for subcortical and cortical brain structures^1^. Note that the imaging data of three mTLE patients with seizure foci in the right hemisphere were left-right flipped before the preprocessing, to match the ipsi- and contra-lateral concept of the analysis. The terms “ipsilateral” and “contralateral” hemispheres stand, respectively for the hemisphere of seizure onset and the hemisphere opposite the side of seizure onset.

### Functional Connectivity Analysis

**Figure 1:**
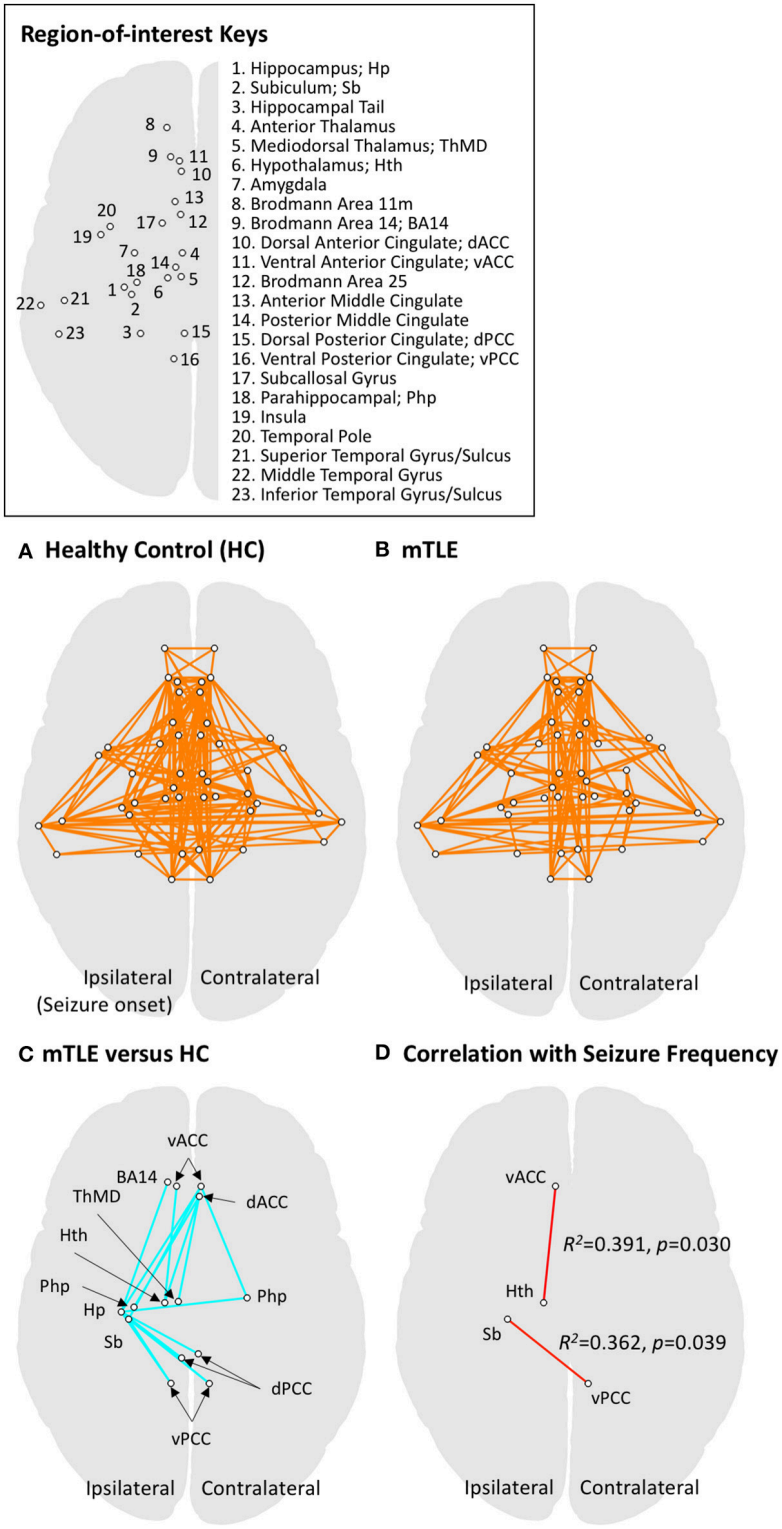
Functional connectivity results for regions of interest (ROIs) in the limbic system and temporal lobe. ROIs in limbic and temporal lobe areas were investigated using network-level analysis (also see Table 3 for details on defining ROIs). **(A)** The graphical representation of functional networks in limbic and temporal lobe regions for the healthy controls (HC), derived by one-sample *t*-test to the zero mean with a threshold level at *p*_*FWER*−*corrected*_ < 0.001. Circles connected by a line represent a pair of ROI nodes and their functional connection. Anatomical labels for the nodes (circles) are described in the upper right panel. **(B)** The graphical representation of functional networks in drug-resistant medial temporal lobe epilepsy (mTLE). The same statistical approach to panel A was applied. The mTLE patients showed decreased limbic functional connectivity relative to the HC group. The functional connectivity among limbic networks and temporal lobe regions, however, showed less change compared to intra-limbic connectivity changes. **(C)** Group difference between functional networks in HC and mTLE groups. A two-sample *t*-test was performed with a threshold level at *p*_*FWER*−*corrected*_ < 0.01. The intra-limbic connectivity between ipsilateral hippocampus, subiculum, hypothalamus, mediodorsal thalamus, ventromedial prefrontal cortex (BA14), bilateral parahippocampal gyri, ventral anterior, dorsal and ventral posterior cingulate cortices, and contralateral dorsal anterior cingulate cortex are decreased in the mTLE group. **(D)** The functional networks significantly correlated with seizure frequency in mTLE patients at a threshold level of *p* < 0.05. Functional connectivity between ipsilateral ventral anterior cingulate cortex and hypothalamus, and that between ipsilateral subiculum and contralateral ventral posterior cingulate cortex showed significant correlation with seizure frequency. The ROI pairs (circles) in limbic system, showing significant difference in functional connectivity between mTLE and HC groups are also summarized in the Table 4.

For the functional network analysis (16, 17), masks for regions-of-interest (ROIs) in the limbic system and temporal lobe structures were defined by multiple atlases (18–21), with reference to existing studies of animal seizure models and human patients (22, 23). The entire list of ROIs and the atlas details are presented in Figure 1A and Table 3. The RS FMRI time series were separately averaged in each ROI mask, and then a Pearson correlation matrix between those was calculated as the network data of each individual subject (by a permutation test for 10,000 iterations with random network extents). A total of 253 RSFCs between ROI pairs were calculated for each subject. The group difference graph between healthy control and mTLE groups were also determined by a two-sample *t*-test with a threshold level at the family-wise-error-corrected *p* < 0.01 (16).

**Table 3:** Definition of regions-of-interest (ROIs) in limbic structures and seizure-relevant areas for the network-level analysis.

**ROI index**	**Region**	**Base atlas**	**Remarks**
**HIPPOCAMPAL FORMATION**			
1	Hippocampus (Hp)	FS-Hp	CA1, CA2, CA3, and CA4 were merged into one mask.
2	Subiculum (Sb)	FS-Hp	Subiculum, parasubiculum, and subsubiculum were merged.
3	Hippocampal tail	FS-Hp	
**DIENCEPHALON**			
4	Anterior nuclei of thalamus	Morel-Th	Anterior dorsal, medial, and ventral nuclei were merged.
5	Mediodorsal nucleus of thalamus (ThMD)	Morel-Th	Magnocellular and parvocellular mediodorsal nuclei were merged.
6	Hypothalamus (Hth)	FS-aseg	Hypothalamus is included in the inferior diencephalon mask.
**SUBCORTEX**			
7	Amygdala	FS-aseg	
**VENTROMEDIAL PREFRONTAL CORTEX (VmPFC)**			
8	Brodmann area 11m	MNI-VmPFC	
9	Brodmann area 14 (BA14)	MNI-VmPFC	BA14c, BA14m, BA14r, and BA14rr were merged.
**CINGULATE CORTEX**			
10	Dorsal anterior (dACC)	MNI-VmPFC	Brodmann area 24
11	Ventral anterior (vACC)	MNI-VmPFC	Brodmann area 32
12	Subgenual anterior (BA25)	MNI-VmPFC	Brodmann area 25
13	Anterior middle	FS-a2009s	
14	Posterior middle	FS-a2009s	
15	Dorsal posterior (dPCC)	FS-a2009s	
16	Ventral posterior (vPCC)	FS-a2009s	
17	Subcallosal gyrus	FS-a2009s	
**OTHER CORTICAL AREAS**			
18	Parahippocampal gyrus (Php)	FS-a2009s	
19	Insula	FS-a2009s	
20	Temporal pole	FS-a2009s	
21	Superior temporal gyrus/sulcus	FS-a2009s	
22	Middle temporal gyrus	FS-a2009s	
23	Inferior temporal gyrus/sulcus	FS-a2009s	

*FS-HP, -aseg, -a2009s are FreeSurfer templates for hippocampal subfields, automatic segmentation, and cortical parcellation, respectively. Morel-Th is the reconstructed template image of Morel histological thalamic atlas, and MNI-VmPFC is the atlas for ventromedial prefrontal cortex of the Montreal Neurological Institute*.

### Correlation Analysis With Clinical Scores

We selected ROI pairs with significant group difference in their RSFC, and then performed a linear regression analysis to find correlations between functional connectivity of those selected ROI pairs and the seizure frequency for mTLE patients. *R*^2^ values were obtained by the linear regression function of MATLAB (version 2016a, The MathWorks, Inc., Natick, MA.).

## Results

### Functional Connectivity Difference Between mTLE and Healthy Control Groups

A total of 253 connections between ROI pairs were considered for group comparisons, and significant connectivity at the threshold level of p_FWE−corrected_ < 0.001 was observed in 17 and 13% of the total ROI pairs in the healthy control and mTLE group, respectively (Figures 1A,B). The mTLE patients showed decreased functional connectivity in the 17 ROI pairs of the limbic system, relative to the healthy control group, by a two-sample *t*-test with the default threshold level of the NBS tool (p_FWE−corrected_ < 0.01). Specifically, the mTLE groups showed diminished intra-limbic connectivity between ipsilateral hippocampus, subiculum, hypothalamus, mediodorsal thalamus, ventromedial prefrontal cortex (BA14), bilateral parahippocampal gyri, ventral anterior, dorsal and ventral posterior cingulate cortices, and contralateral dorsal anterior cingulate cortex (Figures 1C). The functional connectivity between limbic structures and temporal lobe regions showed less change compared to intra-limbic connectivity changes.

### Correlations Between RSFC and Clinical Scores

Two out of 17 limbic ROI pairs, had a significant correlation between their RSFCs and seizure frequency at the threshold level of *p* < 0.05: ipsilateral ventral anterior cingulate cortex and hypothalamus (*R*^2^ = 0.391, *p* = 0.030) and ipsilateral subiculum and contralateral ventral posterior cingulate cortex (*R*^2^ = 0.362, *p* = 0.039) (Figure 1C). The results of group comparisons and their correlation with seizure frequency are summarized in the Table 4 and Figure 1D.

**Table 4:** The ROI pairs (circles) in limbic system, showing significant difference in functional connectivity between mTLE and HC groups.

**Node pair**		***t*-value**	***p*-value (two-tailed)**	**Correlation with seizure frequency**
Hippocampus (ipsilateral)	- vACC (contralateral)	−5.60	< 0.0001	Not significant
	- vPCC (ipsilateral)	−5.46	< 0.0001	Not significant
	- vPCC (contralateral)	−5.37	< 0.0001	Not significant
	- dPCC (ipsilateral)	−6.41	< 0.0001	Not significant
	- dPCC (contralateral)	−4.11	0.0005	Not significant
	- Ventromedial prefrontal cortex (BA14, ipsilateral)	−3.96	0.0007	Not significant
	- Parahippocampal gyrus (contralateral)	−3.30	0.0033	Not significant
Subiculum (ipsilateral)	- vACC (contralateral)	−3.64	0.0014	Not significant
	- vPCC (ipsilateral)	−3.65	0.0014	Not significant
	- vPCC (contralateral)	−3.77	0.0011	*R^2^* = 0.391, *p* = 0.030
	- dPCC (ipsilateral)	−4.31	0.0003	Not significant
Hypothalamus (ipsilateral)	- vACC (ipsilateral)	−3.72	0.0012	*R^2^* = 0.362, *p* = 0.039
	- dACC (contralateral)	−3.67	0.0013	Not significant
Mediodorsal Thalamus (ipsilateral)	- dACC (contralateral)	−3.94	0.0007	Not significant
Parahippocampal gyrus (ipsilateral)	- vACC (contralateral)	−3.11	0.0051	Not significant
	- dACC (contralateral)	−4.82	< 0.0001	Not significant
Parahippocampal gyrus (contralateral)	- vACC (contralateral)	−3.36	0.0028	Not significant

## Discussion

In this study, we compared RS FC in patients with mTLE and healthy controls. Using network-based statistics, we identified connectivity changes associated with mTLE and with seizure frequency in mTLE. RS FC within the limbic system decreased in mTLE, more so than limbic—temporal connectivity. These network abnormalities primarily affected the hemisphere ipsilateral to the seizure focus. Seizure frequency correlated with RS FC between ipsilateral hypothalamus and ventral anterior cingulate cortex and between ipsilateral subiculum and contralateral posterior cingulate cortex.

Our findings are consistent with the observed decrease in functional connectivity between limbic areas in mTLE patients, which is particularly pronounced in the hemisphere ipsilateral to the seizure focus (22, 23). It is possible that the decrease in functional connectivity reflects underlying limbic pathology in the mTLE group through hypothalamus-to-mediodorsal-thalamus and hippocampus/subiculum-to-cingulate-cortex pathways (24, 25). These changes would be consistent with the affective, memory, and cognitive/behavioral symptoms experienced by mTLE patients (2).

It is also possible that the decrease in limbic RS FC is compensatory. It may be speculated that decreasing functional connectivity ipsilateral to the seizure focus helps limit the participation of the abnormal temporal lobe in limbic circuitry, thereby preserving some measure of normal limbic function. The findings reported here for FMRI connectivity are consistent with studies using intracranial electrocorticography, which show that brain regions generating seizures are functionally isolated from surrounding brain regions (26, 27).

It is unclear why connectivity pairs in the cingulate cortex and hypothalamus and in the cingulate cortex and subiculum emerged as associated with seizure frequency. Previous network studies have focused on the clinical outcome of seizure freedom and not seizure frequency. One study of seizure propagation networks showed that seizure freedom associated with decreased functional connectivity in bilateral midline structures including the precuneus and midcingulate (28). This finding, together with our observation that select cingulate connectivity features are associated with seizure frequency, may implicate midline connectivity as an overall indicator of limbic network health. Importantly, without larger studies pooling subjects with similar disease courses, this proposal is purely speculative.

With the limbic system acting as a centerpoint for a range of cognitive and seizure-related processes, broader network changes may help explain the role of specific limbic regions in epilepsy (29). A pilot study of functional connectivity in deep brain stimulation (DBS) for epilepsy has suggested that patients who respond to DBS at the anterior nucleus of the thalamus have increased thalamic connectivity to the DMN (30). Intrinsic DMN connectivity increases prior to inter-ictal epileptiform discharges in TLE before returning to its baseline, decreased connectivity after the discharge (31). Further study of how paired connectivity features interact with other resting-state functional networks is warranted.

The primary limitation of this study was the small sample size. We did not conduct structured psychiatric interviews for this study and only retrospectively collected psychiatric histories from the medical record. Future efforts building on the present work will include prospective and comprehensive structured psychiatric assessments. A deeper understanding of the cause and nature of limbic connectivity in patients with mTLE may prove important in treating mTLE and the comorbidities associated with limbic network dysfunction. Also, we flipped the EPI data for left-handed subjects, but there are reports showing that mTLE patients have different behaviors regarding to the anatomical and functional features on the laterality (32–34). To observe more details on the laterality of mTLE connectivity, the further investigation with larger cohorts is required.

In conclusion, we identified functional connectivity changes associated with mTLE and cingulate and limbic network connectivity abnormalities that correlated with increased seizure frequency.

## Ethics Statement

All participants provided written, informed consent in accordance with research protocols approved by the institutional review board of Mayo Clinic.

## Author Contributions

HJ, KW, IB, and GW: design and conceptualization of the study. HJ, DK-J, PC, EB, and GW: analysis and interpretation of the data. HJ, IB, and DK-J: drafting the manuscript for intellectual content. PC, DJ, EB, GW, and IB: revising the manuscript for intellectual content. KW and DJ: major role in the acquisition of data.

### Conflict of Interest Statement

The authors declare that the research was conducted in the absence of any commercial or financial relationships that could be construed as a potential conflict of interest.

## Footnotes

^1^Aside from the visual inspection to assess thalamic atrophy, recent studies using quantitative MRI analyses have reported global as well as segmental thalamic atrophy in TLE [see (15) for review].”

## References

[1] Papez JW. A proposed mechanism of emotion. 1937. J Neuropsychiatry Clin Neurosci. (1995) 7:103–12. 10.1176/jnp.7.1.103 7711480

[2] Travers RF. Limbic epilepsy. J R Soc Med. (1991) 84:454–6. 10.1177/014107689108400804 1909369 PMC1293371

[3] Finegersh A Avedissian C Shamim S Dustin I Thompson PM Theodore WH. Bilateral hippocampal atrophy in temporal lobe epilepsy: effect of depressive symptoms and febrile seizures. Epilepsia. (2011) 52:689–97. 10.1111/j.1528-1167.2010.02928.x 21269286 PMC3071425

[4] Blumenfeld H. Impaired consciousness in epilepsy. Lancet Neurol. (2012) 11:814–26. 10.1016/S1474-4422(12)70188-6 22898735 PMC3732214

[5] Jefferys JGR Jiruska P De Curtis M Avoli M Limbic Network Synchronization and Temporal Lobe Epilepsy. In: Th JL Noebels M Avoli MA Rogawski RW Olsen AV Delgado-Escueta editors. Jasper's Basic Mechanisms of the Epilepsies. Bethesda, MD: National Institutes of Health (2012). 22787650

[6] Hermann BP Seidenberg M Schoenfeld J Davies K. Neuropsychological characteristics of the syndrome of mesial temporal lobe epilepsy. Arch Neurol. (1997) 54:369–76. 10.1001/archneur.1997.00550160019010 9109737

[7] Kanner AM. Mood disorder and epilepsy: a neurobiologic perspective of their relationship. Dialogues Clin Neurosci. (2008) 10:39–45. Available online at: https://www.dialogues-cns.org/contents-10-1/18472483 10.31887/DCNS.2008.10.1/amkanner PMC3181864

[8] Chiang S Haneef Z. Graph theory findings in the pathophysiology of temporal lobe epilepsy. Clin Neurophysiol. (2014) 125:1295–305. 10.1016/j.clinph.2014.04.004 24831083 PMC4281254

[9] Haneef Z Chiang S. Clinical correlates of graph theory findings in temporal lobe epilepsy. Seizure. (2014) 23:809–18. 10.1016/j.seizure.2014.07.004 25127370 PMC4281255

[10] He X Doucet GE Pustina D Sperling MR Sharan AD Tracy JI. Presurgical thalamic “hubness” predicts surgical outcome in temporal lobe epilepsy. Neurology. (2017) 88:2285–93. 10.1212/WNL.0000000000004035 28515267 PMC12477986

[11] Park C-H Choi YS Kim HJ Chung H-K Jung A-R Yoo JH. Interactive effects of seizure frequency and lateralization on intratemporal effective connectivity in temporal lobe epilepsy. Epilepsia. (2018) 59:215–25. 10.1111/epi.13951 29205291

[12] Jo HJ Saad ZS Simmons WK Milbury LA Cox RW. Mapping sources of correlation in resting state FMRI, with artifact detection and removal. Neuroimage. (2010) 52:571–82. 10.1016/j.neuroimage.2010.04.246 20420926 PMC2897154

[13] Gotts SJ Jo HJ Wallace GL Saad ZS Cox RW Martin A. Two distinct forms of functional lateralization in the human brain. Proc Natl Acad Sci USA. (2013) 110:E3435–44. 10.1073/pnas.1302581110 23959883 PMC3767540

[14] Jo HJ Gotts SJ Reynolds RC Bandettini PA Martin A Cox RW. Effective preprocessing procedures virtually eliminate distance-dependent motion artifacts in resting state FMRI. J Appl Mathemat. (2013) 2013:935154. 10.1155/2013/935154 24415902 PMC3886863

[15] Bonilha L Keller SS. Quantitative MRI in refractory temporal lobe epilepsy: relationship with surgical outcomes. Quant Imaging Med Surg. (2015) 5:204–24. 10.3978/j.issn.2223-4292.2015.01.01 25853080 PMC4379322

[16] Zalesky A Fornito A Bullmore ET. Network-based statistic: identifying differences in brain networks. Neuroimage. (2010) 53:1197–207. 10.1016/j.neuroimage.2010.06.041 20600983

[17] Chiang S Stern JM Engel J Jr Levin HS Haneef Z. Differences in graph theory functional connectivity in left and right temporal lobe epilepsy. Epilepsy Res. (2014) 108:1770–81. 10.1016/j.eplepsyres.2014.09.023 25445238 PMC5013648

[18] Destrieux C Fischl B Dale A Halgren E. Automatic parcellation of human cortical gyri and sulci using standard anatomical nomenclature. Neuroimage. (2010) 53:1–15. 10.1016/j.neuroimage.2010.06.010 20547229 PMC2937159

[19] Krauth A Blanc R Poveda A Jeanmonod D Morel A Szekely G. A mean three-dimensional atlas of the human thalamus: generation from multiple histological data. Neuroimage. (2010) 49:2053–62. 10.1016/j.neuroimage.2009.10.042 19853042

[20] Mackey S Petrides M. Architecture and morphology of the human ventromedial prefrontal cortex. Eur J Neurosci. (2014) 40:2777–96. 10.1111/ejn.12654 25123211

[21] Iglesias JE Augustinack JC Nguyen K Player CM Player A Wright M. A computational atlas of the hippocampal formation using *ex vivo*, ultra-high resolution MRI: application to adaptive segmentation of *in vivo* MRI. Neuroimage. (2015) 115:117–37. 10.1016/j.neuroimage.2015.04.042 25936807 PMC4461537

[22] Bharath RD Sinha S Panda R Raghavendra K George L Chaitanya G. Seizure frequency can alter brain connectivity: evidence from resting-state fMRI. AJNR Am J Neuroradiol. (2015) 36:1890–8. 10.3174/ajnr.A4373 26294642 PMC7965029

[23] Gill RS Mirsattari SM Leung LS. Resting state functional network disruptions in a kainic acid model of temporal lobe epilepsy. Neuroimage Clin. (2017) 13:70–81. 10.1016/j.nicl.2016.11.002 27942449 PMC5133653

[24] Bertram EH Mangan PS Zhang D Scott CA Williamson JM. The midline thalamus: alterations and a potential role in limbic epilepsy. Epilepsia. (2001) 42:967–78. 10.1046/j.1528-1157.2001.042008967.x 11554881

[25] Maccotta L He BJ Snyder AZ Eisenman LN Benzinger TL Ances BM. Impaired and facilitated functional networks in temporal lobe epilepsy. Neuroimage Clin. (2013) 2:862–72. 10.1016/j.nicl.2013.06.011 24073391 PMC3777845

[26] Warren CP Hu S Stead M Brinkmann BH Bower MR Worrell GA. Synchrony in normal and focal epileptic brain: the seizure onset zone is functionally disconnected. J Neurophysiol. (2010) 104:3530–9. 10.1152/jn.00368.2010 20926610 PMC3007634

[27] Burns SP Santaniello S Yaffe RB Jouny CC Crone NE Bergey GK. Network dynamics of the brain and influence of the epileptic seizure onset zone. Proc Natl Acad Sci USA. (2014) 111:E5321–30. 10.1073/pnas.1401752111 25404339 PMC4267355

[28] Morgan VL Englot DJ Rogers BP Landman BA Cakir A Abou-Khalil BW. Magnetic resonance imaging connectivity for the prediction of seizure outcome in temporal lobe epilepsy. Epilepsia. (2017) 58:1251–60. 10.1111/epi.13762 28448683 PMC5498250

[29] Widjaja E Zamyadi M Raybaud C Snead OC Doesburg SM Smith ML. Disrupted global and regional structural networks and subnetworks in children with localization-related epilepsy. Am J Neuroradiol. (2015) 36:1362–8. 10.3174/ajnr.A4265 25742984 PMC7965281

[30] Middlebrooks EH Grewal SS Stead M Lundstrom BN Worrell GA Van Gompel JJ. Differences in functional connectivity profiles as a predictor of response to anterior thalamic nucleus deep brain stimulation for epilepsy: a hypothesis for the mechanism of action and a potential biomarker for outcomes. Neurosurg Focus. (2018) 45:E7. 10.3171/2018.5.FOCUS18151 30064322

[31] Lopes R Moeller F Besson P Ogez F Szurhaj W Leclerc X. Study on the relationships between intrinsic functional connectivity of the default mode network and transient epileptic activity. Front Neurol. (2014) 5:201. 10.3389/fneur.2014.00201 25346721 PMC4193009

[32] Bonilha L Rorden C Halford JJ Eckert M Appenzeller S Cendes F. Asymmetrical extra-hippocampal grey matter loss related to hippocampal atrophy in patients with medial temporal lobe epilepsy. J Neurol Neurosurg Psychiatr. (2007) 78:286–94. 10.1136/jnnp.2006.103994 17012334 PMC2117646

[33] Besson P Dinkelacker V Valabregue R Thivard L Leclerc X Baulac M. Structural connectivity differences in left and right temporal lobe epilepsy. Neuroimage. (2014) 100:135–44. 10.1016/j.neuroimage.2014.04.071 24814212

[34] De Campos BM Coan AC Lin Yasuda C Casseb RF Cendes F. Large-scale brain networks are distinctly affected in right and left mesial temporal lobe epilepsy. Hum Brain Mapp. (2016) 37:3137–52. 10.1002/hbm.23231 27133613 PMC5074272

